# Augmentation with an ovine forestomach matrix scaffold improves histological outcomes of rotator cuff repair in a rat model

**DOI:** 10.1186/s13018-015-0303-8

**Published:** 2015-10-20

**Authors:** Matthew Street, Ashvin Thambyah, Michael Dray, Satya Amirapu, Donna Tuari, Karen E Callon, Julie D McIntosh, Kristina Burkert, P Rod Dunbar, Brendan Coleman, Jillian Cornish, David S Musson

**Affiliations:** Department of Medicine, The University of Auckland, Private Bag 92019, Auckland, 1142 New Zealand; Faculty of Engineering, University of Auckland, Auckland, 1142 New Zealand; Waikato District Health Board, Waikato Hospital, Hamilton, 3204 New Zealand; Department of Anatomy with Radiology, The University of Auckland, Auckland, 1142 New Zealand; School of Biological Sciences, The University of Auckland, Auckland, 1142 New Zealand; Maurice Wilkins Centre, University of Auckland, Private Bag 92014, Auckland, New Zealand; Department of Orthopaedics, Middlemore Hospital, Private Bag 93311, Auckland, New Zealand

**Keywords:** Rotator Cuff, Arthroplasty, Augmentation, Biomaterial, Tissue engineering

## Abstract

**Background:**

Rotator cuff tears can cause significant pain and functional impairment. Without surgical repair, the rotator cuff has little healing potential, and following surgical repair, they are highly prone to re-rupture. Augmenting such repairs with a biomaterial scaffold has been suggested as a potential solution. Extracellular matrix (ECM)-based scaffolds are the most commonly used rotator cuff augments, although to date, reports on their success are variable. Here, we utilize pre-clinical in vitro and in vivo assays to assess the efficacy of a novel biomaterial scaffold, ovine forestomach extracellular matrix (OFM), in augmenting rotator cuff repair.

**Methods:**

OFM was assessed in vitro for primary tenocyte growth and adherence, and for immunogenicity using an assay of primary human dendritic cell activation. In vivo, using a murine model, supraspinatus tendon repairs were carried out in 34 animals. Augmentation with OFM was compared to sham surgery and unaugmented control. At 6- and 12-week time points, the repairs were analysed biomechanically for strength of repair and histologically for quality of healing.

**Results:**

OFM supported tenocyte growth in vitro and did not cause an immunogenic response. Augmentation with OFM improved the quality of healing of the repaired tendon, with no evidence of excessive inflammatory response. However, there was no biomechanical advantage of augmentation.

**Conclusions:**

The ideal rotator cuff tendon augment has not yet been identified or clinically implemented. ECM scaffolds offer a promising solution to a difficult clinical problem. Here, we have shown improved histological healing with OFM augmentation. Identifying materials that offset the poorer mechanical properties of the rotator cuff post-injury/repair and enhance organised tendon healing will be paramount to incorporating augmentation into surgical treatment of the rotator cuff.

## Introduction

Rotator cuff tears are a common cause of significant shoulder pain and functional impairment and are the most common cause of shoulder-related disability [1]. They are estimated to affect 22 % of the general population, and the prevalence significantly increases with age [2]. Furthermore, with our increasing and ageing population, the incidence of rotator cuff tears requiring surgical intervention is steadily increasing [3].

Due to the avascular and acellular nature of the rotator cuff, healing potential is limited without surgical repair [4–6]. In a rat model of rotator cuff tears, histological analysis showed that 78 % of tendons had incomplete closure of the defect site at 12 weeks post defect creation, and those defects that were fully closed had disorganised tissue inferior to that of the normal tendon [4]. Even after surgical repair, healing is suboptimal. In human patients over the age of 65, only 43 % of patients showed evidence of healing in a computed tomographic arthrogram or magnetic resonance imaging scans 18 months after arthroscopic repair of full thickness rotator cuff tears, while 86 % of patients under 65 years of age showed evidence of healing [7]. Furthermore, re-tear rates are reported to be as high as 41 % for double row arthroscopic repairs and 69 % for single row repairs of large full thickness rotator cuff tears [8, 9].

Recently, biological augmentation with tissue-engineered grafts has been suggested as a method for improving healing outcomes in grades 3 and 4 tears (repairable, full thickness, small to large tears) and as an interposition graft for grade 5 tears (large retracted tears that are unable to be apposed) [10]. These grafts can be used either as an onlay (reinforcement) augmentation, where the graft is placed over the repaired tendon, or as an intercalary (interpositional) augmentation, where the graft is placed between the bone and retracted tendon.

Augmentation has two potential benefits: (1) providing mechanical support and offloading the tendon at the time of initial repair and (2) improving the rate and/or quality of healing [11]. Therefore, the ideal graft would achieve both of these benefits with the added parameters that it has good suture retention properties and is immunologically inert and fully degradable in a biological setting.

Extracellular matrix (ECM)-based scaffolds are currently the most commonly used rotator cuff augments [11]. Most components of ECM are homogenous among different species allowing properly processed ECM xenografts to be used with minimal anti-host inflammation [12, 13]. Furthermore, ECM has a complex three-dimensional microstructure, involving structural proteins such as collagen, fibronectin and laminin, glycosaminoglycans (GAGs) and bioactive growth factors. Combined, these allow for a degree of mechanical support, but more importantly, provide host cell attachment sites and a reservoir of growth factors that encourage cell migration, proliferation and differentiation while also modulating angiogenesis and immune response [12].

Recently, clinical confidence in the use of ECM scaffolds received a significant setback when a porcine non-cross-linked SIS (Restore™) was associated with hypersensitivity reactions in 20–30 % of patients [14, 15]. It has been suggested that this reaction was secondary to a large amount of foreign DNA retained in Restore™ and led to the product being no longer recommended for use by the American Academy of Orthopaedic Surgeons [15, 16]. Therefore, all potential scaffolds need to be thoroughly tested prior to clinical use and closely monitored following commercial release.

Decellularized ovine forestomach matrix (OFM) is an ECM scaffold produced from the lamina propria of the ovine forestomach. It is decellularized using proprietary processes that retain the native collagen structure and secondary molecules that associate with ECM [17]. OFM is used clinically as a dermal template in the treatment of chronic wounds [18–20], can act as a template for tissue engineered skin grafts [21] and has also been used successfully as split thickness skin grafts [22].

Through the studies presented here, we aim to assess the healing potential of OFM as a novel rotator cuff augmentation graft by thorough in vitro evaluation and assessment in a rat supraspinatus defect model. Clinically, rotator cuff augmentation could lead to reduced re-tear rates, earlier mobilisation of the shoulder and thus reduced joint stiffness and ultimately improved post-operative function.

## Methods

### Ethical approval

The surgical procedure and isolation of primary rat tenocytes were carried out in accordance with the Institutional Animal Ethics Committee. Peripheral blood mononuclear cells were purified from healthy human volunteer blood and collected according to a protocol approved by the Institutional Human Participants Ethics Committee.

### Tenocyte cell culture

Primary rat tenocytes were isolated from tendon fascicles of mature female Wistar rats, as previously described [23]. Briefly, tendon was roughly chopped and digested in dispase and collagenase (both 0.5 mg/mL from Sigma-Aldrich, USA) in Dulbecco’s modified Eagle’s medium-F12 (DMEM-F12) with 10 % fetal bovine serum (FBS) at 37 °C until all the ECM had been digested. The cell suspension was passed through a cell strainer, washed and re-suspended in fresh media. Cells were cultured in 75 cm^2^ flasks (Corning Inc., USA) with 10 % FBS/DMEM-F12 and incubated at 37 °C with 5 % CO_2_ until confluent.

### Scaffold preparation

OFM was provided by Mesynthes Ltd. (Auckland, New Zealand) as single-ply, lyophilized biomaterial, terminally sterilized with ethylene oxide. Samples were pre-packaged as 1.9-cm diameter discs or grafts pre-cut to 5 × 10 mm.

### Qualitative analysis of OFM cytocompatibility

Primary rat tenocytes, isolated as above, were seeded onto 1.9-cm diameter discs of OFM in 24-well plates (Greiner Bio-One, Germany) for a period of 1, 7 and 14 days in DMEM-F12 with 5 % FBS at 2.5 × 10^4^ cells/scaffold. At each time point, scaffolds were stained with calcein AM (Invitrogen) to visualise live cells, as previously described [24]. Scaffolds were washed in phosphate-buffered saline (PBS) and stained for 10 min at 37 °C with 2 μM calcein AM in PBS. Scaffolds were washed in fresh PBS and viewed immediately using fluorescent microscopy; representative images were taken to demonstrate cell density across the scaffold.

### Quantitative analysis of OFM cytocompatibility

Primary rat tenocytes, isolated as above, were seeded as described for the qualitative cytocompatibility assay for a period of 1, 7 and 14 days. At each time point, viability was assessed, as previously described [24]. Briefly, 50 μl alamarBlue® (×100, Invitrogen) (5 % final concentration in well) was added to each well for 4 h at 37 °C. Following this, 200 μl of the alamarBlue® conditioned medium was transferred to a 96-well plate (Greiner Bio-One) and fluorescence read using a Synergy 2 multi-detection microplate reader (BioTek Instruments Inc., USA).

### Immunogenicity assay—monocyte-derived dendritic cell activation

Peripheral blood mononuclear cells were purified from healthy human volunteer blood. Monocytes were isolated from peripheral blood mononuclear cells using the Monocyte Isolation Kit II (Miltenyi Biotec, Germany), according to the manufacturer’s protocol. Immature monocyte-derived dendritic cells were prepared by culturing the monocytes for 6 days in RPMI medium supplemented with 10 % FBS, 100 ng/mL granulocyte macrophage-colony stimulating factor (GM-CSF) and 50 ng/mL interleukin-4 (IL-4) (PeproTech, USA).

The immunogenicity of OFM was assessed using immature dendritic cells, as previously described [24]. Cells were plated in 24-well plates containing either 5 ng/mL lipopolysaccharide (LPS; Sigma-Aldrich) or 1.9-cm diameter OFM in RPMI medium with 10 % FBS. The expression of cell surface markers associated with dendritic cell maturation was assessed following 40 h of incubation.

Treated dendritic cells were incubated on ice for 20 min with mouse anti-human antibodies against PE/Cy7-anti-CD80 (clone 2D10), PerCP/Cy5.5 anti-CD83 (clone HB15e) and APC anti-CD86 (clone IT2.2) (all supplied by BioLegend, USA). Excess antibody was removed by washing with PBS containing 1 % FBS. Cell surface markers were analysed by flow cytometry using the FACS ARIA II (BD Biosciences), and data analysis was performed using FloJo (version 7.6) (BD Biosciences).

### In vivo assessment

Male Sprague-Dawley rats (*n* = 34), aged greater than 12 weeks and weighing greater than 350 g, underwent surgery. Rats were checked for general health and randomly distributed into three weight-matched groups: (1) sham surgery (approach to supraspinatus only), *n* = 10, (2) unaugmented control (single row supraspinatus repair), *n* = 12 and (3) intervention group (single row repair augmented with OFM), *n* = 12.

At least 2 h prior to surgery, rats received a subcutaneous injection of the non-steroidal anti-inflammatory drug (NSAID) Rimadyl (10 μL/g). For anaesthetic induction, rats were placed in a sealed rodent induction box with 5 % isoflurane and 2 L O_2_. Anaesthesia was maintained during surgery with 2.5 % isoflurane and 2 L O_2_ through a specialised nose cone. A 2.5-mL subcutaneous injection of normal saline was administered immediately after induction.

**Fig. 1:**
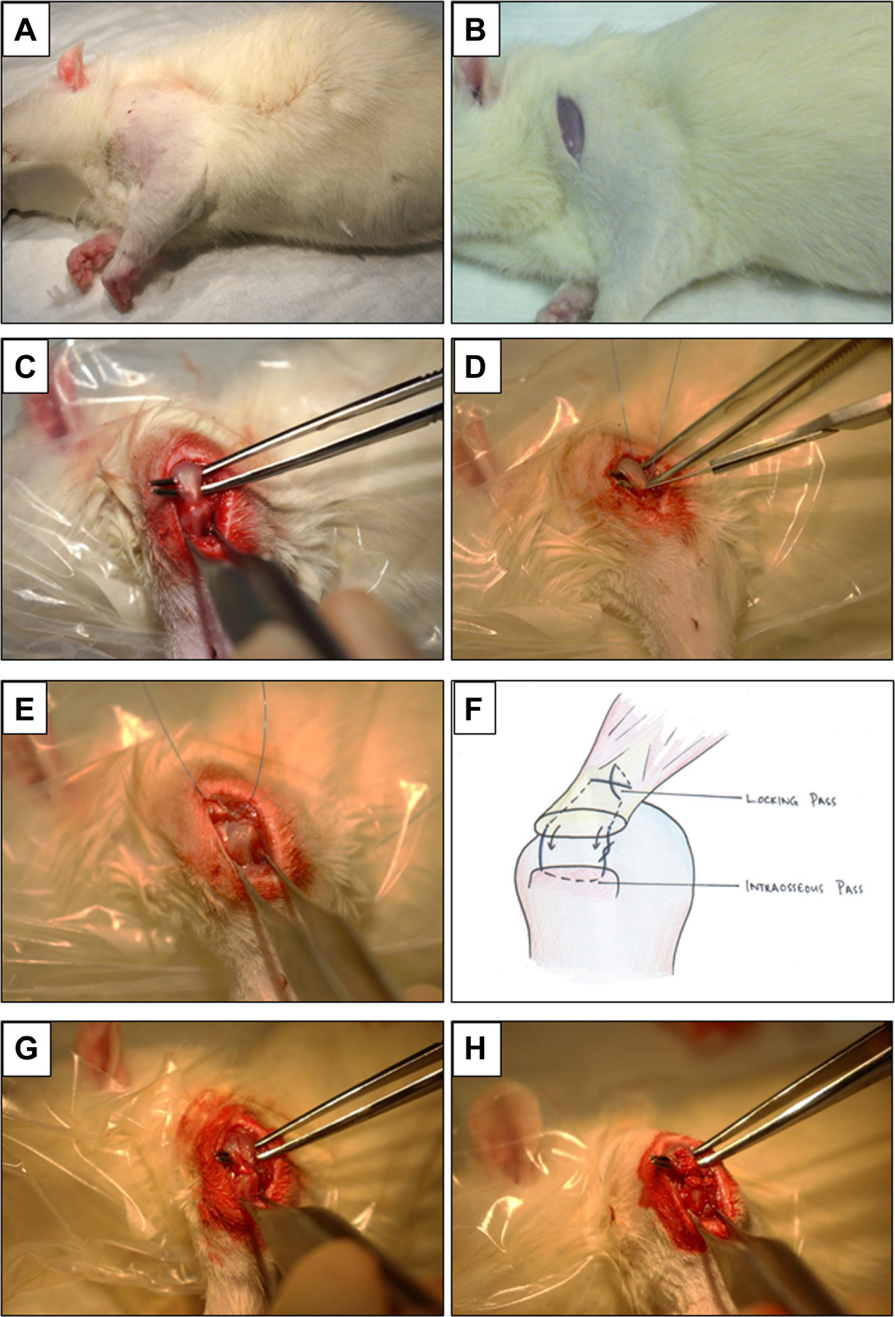
Intraoperative guide to in vivo surgery. Micrographs representing each stage of the operative procedure including the preparation and positioning of the shoulder (**a**), creating an incision over the humeral head (**b**), isolating the exposed supraspinatus (**c**), transecting the supraspinatus from the humeral head (**d**), removing residual soft tissue from the humeral head (**e**), the modified Mason-Allen suture repair (**f**) and the unaugmented and augmented supraspinatus tendons (**g**, **h**)

The forelimb was prepared by shaving and washing with 2 % chlorhexidine and 70 % ethanol, followed by a sterile drape (Fig. 1a). An approximately 2-cm-longitudinal incision was made, centred over the glenohumeral joint, extending proximally along the belly of supraspinatus and distally along shaft of humerus (Fig. 1b). The middle belly of deltoid was incised proximally, in line with muscle fibres, down to the lateral aspect of the proximal humeral shaft. The acromio-clavicular joint was incised. The supraspinatus was easily identified through this approach (Fig. 1c). A 5-0 prolene stay suture was passed through the distal supraspinatus. The tendon was incised at its insertion onto the greater tuberosity of the humerus (Fig. 1d). The insertion site was debrided of any residual soft tissue (Fig. 1e).

In the unaugmented control group, supraspinatus repair was carried out using the 5-0 prolene stay suture and a modified Mason-Allen suture technique (Fig. 1f). The cut end of the tendon was approximated to the bony insertion, and the suture ends were tied with a surgeon’s knot (Fig. 1g). In the intervention group, the OFM scaffolds (5 × 10 mm) were rehydrated with sterile saline, then overlaid longitudinally on the superficial aspect of the tendon-bone insertion and incorporated into the suture repair (passes 2-5) (Fig. 1h).

Following repair, the deltoid and coraco-acromial arch were lightly approximated with two interrupted 2-0 vicryl sutures. Skin closure was carried out with a running subcuticular 4-0 monocryl suture with buried knots at either ends. After closure, 0.4 mL of Marcain (1.25 mg/mL solution) local anaesthetic was infiltrated around the operation site.

Rats were closely monitored immediately following surgery. Once sufficiently recovered from the anaesthetic, they were housed singularly and transferred to a warming cabinet for one night. Rimadyl (10 μL/g) and 2-mL normal saline were administered subcutaneously twice daily for 48 h post-operation. Rats were weighed daily and checked for signs of illness, pain or distress twice daily for the first 48 h post-operation. Following this, they were weighed and checked once daily until 14 days post-operation. They were then weighed and checked on a weekly basis.

At either 6 or 12 weeks post-operatively, rats were humanely sacrificed by CO_2_ inhalation. The left supraspinatus tendon, attached to the whole humerus, was immediately excised and either placed in formalin for histological analysis or wrapped in PBS soaked gauze and stored at −20 °C for later biomechanical analysis.

### Biomechanical analysis

Excised shoulders were defrosted in a 37 °C water bath and kept hydrated with H_2_O spray throughout testing. The muscle fibres of the supraspinatus were removed by gentle scraping, leaving only the distal tendon attached to the humerus. Suture material was removed to allow testing of the repaired tendon alone.

Using a specially modified clamp, the humerus and the tendon were positioned in an Instron machine, with the tendon at 45° relative to the humerus, to apply load in a functional position. Specimens underwent a 10-cycle preconditioning phase (0.1 to 0.5 N at a rate of 1 %/s), followed by a stress relaxation phase (6 % strain, at a rate of 5 %/s (0.575 mm/s), followed by 10 min of relaxation, then ramp to failure at a rate of 0.3 %/s. Stiffness, Young’s modulus of elasticity and ultimate load to failure were then calculated. For each treatment group, at each time point (6 and 12 weeks) *n* = 4 samples were tested.

### Histological analysis

Excised shoulders were fixed in 10 % neutral buffered formalin, processed, decalcified in 10 % formic acid/5 % formaldehyde, embedded in paraffin and 5-μm-thick sections taken. Sections were stained with haematoxylin and eosin (H&E) and analysed using both transmitted and polarised light microscopy to evaluate healing. A semi-quantitative grading system was used based on collagen fibre density, collagen fibre orientation, quality of healing at bone-tendon interface, vascularity and presence of inflammatory cells, as described in Table 1. A total was obtained by combining the scores for these five parameters, with a higher score indicating greater healing. Scoring was carried out by a musculoskeletal histopathologist (MD) and an orthopaedic registrar (MS) who were blinded to treatment details and each other’s scores. A minimum of three slides per repair were assessed where the tendon-bone interface could be identified.

**Table 1:** Histological grading system used to determine the rotator cuff healing outcomes

	Collagen fibre density	Collagen fibre orientation	Bone-tendon interface	Vascularity	Inflammation
0	None	None	0–24 % interdigitation	Abundant vascular network	Abundant inflammatory cells
1	Low	Disorganised fibres	25–49 % interdigitation	Moderate vascular network	Moderate inflammatory cells
2	Medium	Moderate alignment	50–75 % interdigitation	Minimal vascular network	Minimal inflammatory cells
3	High	Highly aligned	>75 % interdigitation	No vascular network	No inflammatory cells

### Statistical analysis

Data from both the in vitro and in vivo analyses were analysed using one-way analysis of variance (ANOVA) with post hoc Tukey’s test using GraphPad Prism Software (GraphPad Software, San Diego, CA, USA).

## Results

### Qualitative and quantitative analysis of OFM cytocompatibility

**Fig. 2:**
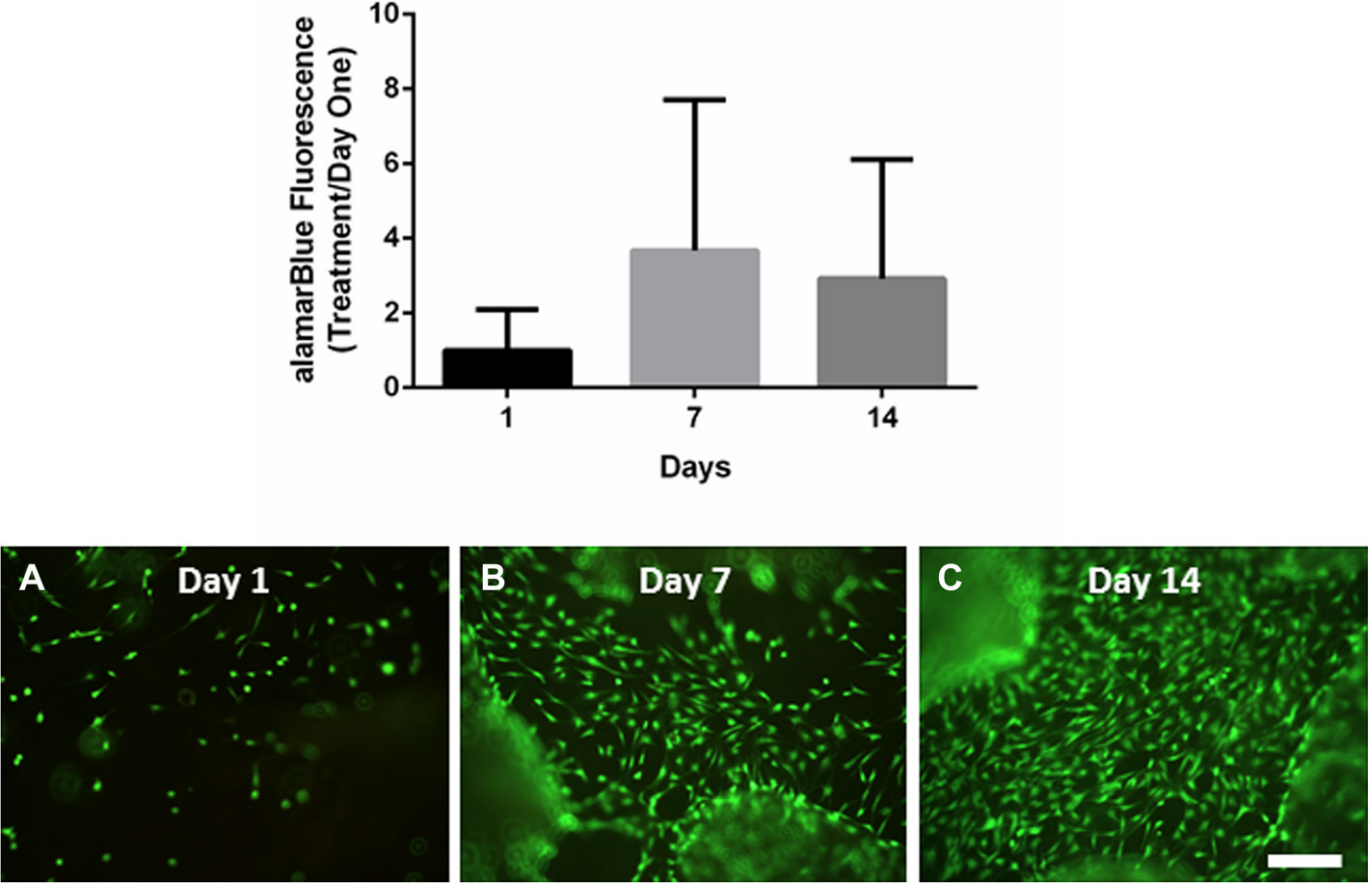
OFM supports primary rat tenocyte growth. Cytocompatibility analysis of primary rat tenocytes cultured on OFM for 1, 7 and 14 days using alamarBlue™ assay and calcein AM fluorescent staining (**a** day 1, **b** day 7, **c** day 14). Scale bar = 200 μm. Observations were performed three times. Data presented are representative of these biological repeats. *N* = 3

Cytocompatibility of OFM was confirmed quantitatively by alamarBlue™ assay of tenocyte viability and qualitatively by calcein AM fluorescent staining of live tenocytes. Quantitative analysis demonstrated that tenocyte cell numbers increase from day 1 to day 7, after which cell numbers change little up to day 14. Cell growth was highly variable over the 14-day culture period, as demonstrated by the large error bars. Qualitative analysis demonstrated that primary rat tenocytes successfully adhered to OFM on day 1 and the number of viable cells increased over the 14-day period (Fig. 2).

### Immunogenicity assay—monocyte-derived dendritic cell activation

The immunogenicity of implantable scaffolds has been strongly correlated to the maturation of antigen-presenting dendritic cells, including the upregulation of dendritic cell surface proteins such as CD80, CD83 and CD86.

**Fig. 3:**
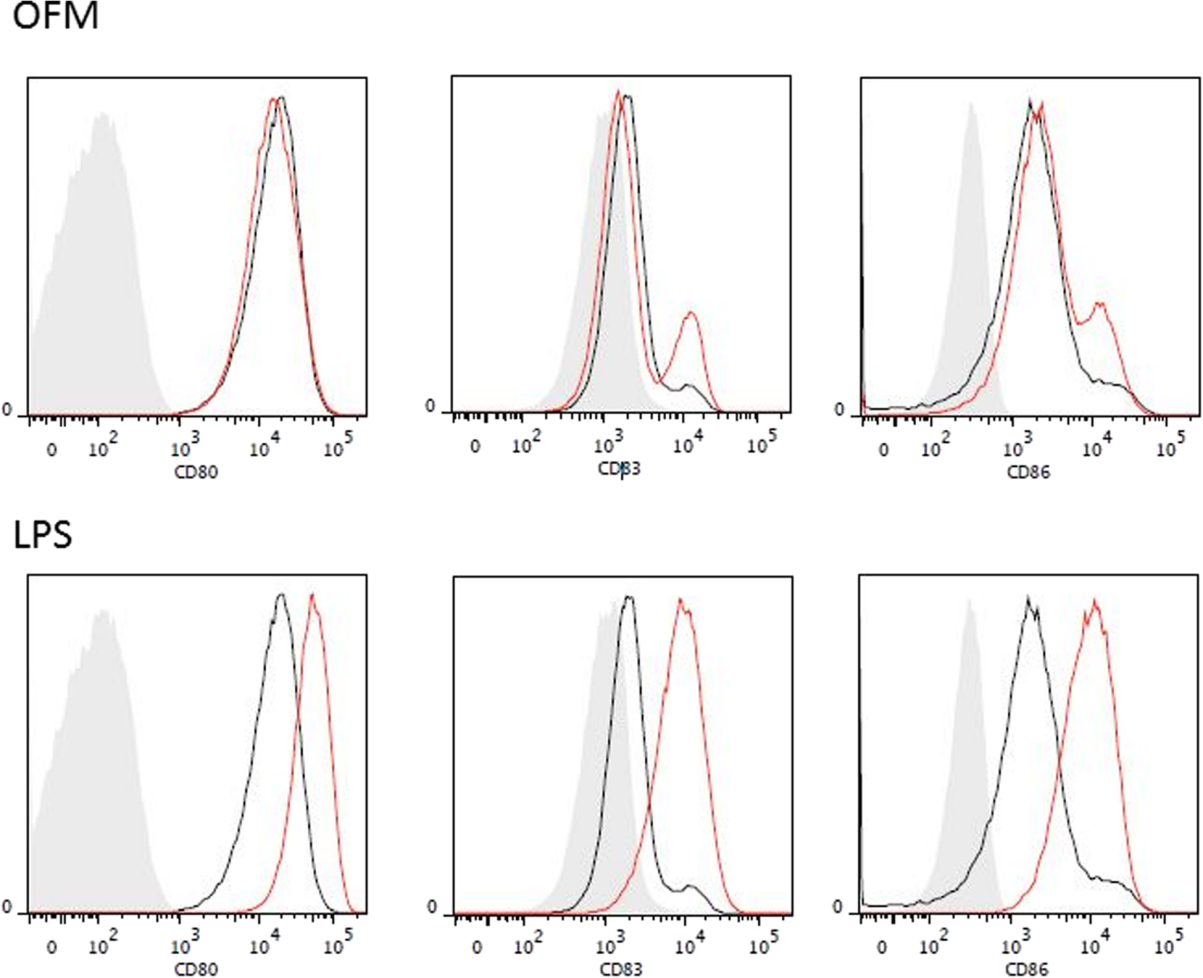
OFM does not promote dendritic cell maturation. Representative FACS plots of CD80, CD86 and CD83 expression, as markers of dendritic cell maturation, following 40 h of culture with OFM and LPS. The *shaded area* represents the unstained control, the *black line* is the untreated control and the *red line* represents the treatment group as indicated. Three different patient samples were analysed, each experiment run in duplicates. Data presented are from one patient as representative for the three patients. *N* = 3

Following 40 h of exposure to OFM, dendritic cell expression levels of CD80, CD83 and CD86 were unchanged compared to the low levels expressed by untreated control dendritic cells (Fig. 3). This was in contrast to dendritic cells exposed to LPS, which had elevated levels of all three maturation markers.

### In vivo assessment

All surgeries were uneventful with no anaesthetic or operative complications. Post operatively, rats recovered rapidly, returning to full use of the operated limb within 48–72 h. All 34 animals survived until the experimental endpoints, and no hypersensitivity reactions were observed. Furthermore, macroscopic inspection of the collected specimens did not reveal any gross infectious or inflammatory changes.

### Biomechanical analysis

An important outcome required when augmenting a rotator cuff repair is for the biomechanical performance of the tendon to replicate its pre-injury state allowing appropriate forces across the repair to aid healing by improving collagen orientation. In order to assess this, the repaired rotator cuffs were biomechanically tested along a functional axis, with stiffness, elasticity and total load to failure measured. Biomechanical analysis revealed no significant differences in stiffness, elasticity or load to failure at either time points.

**Fig. 4:**
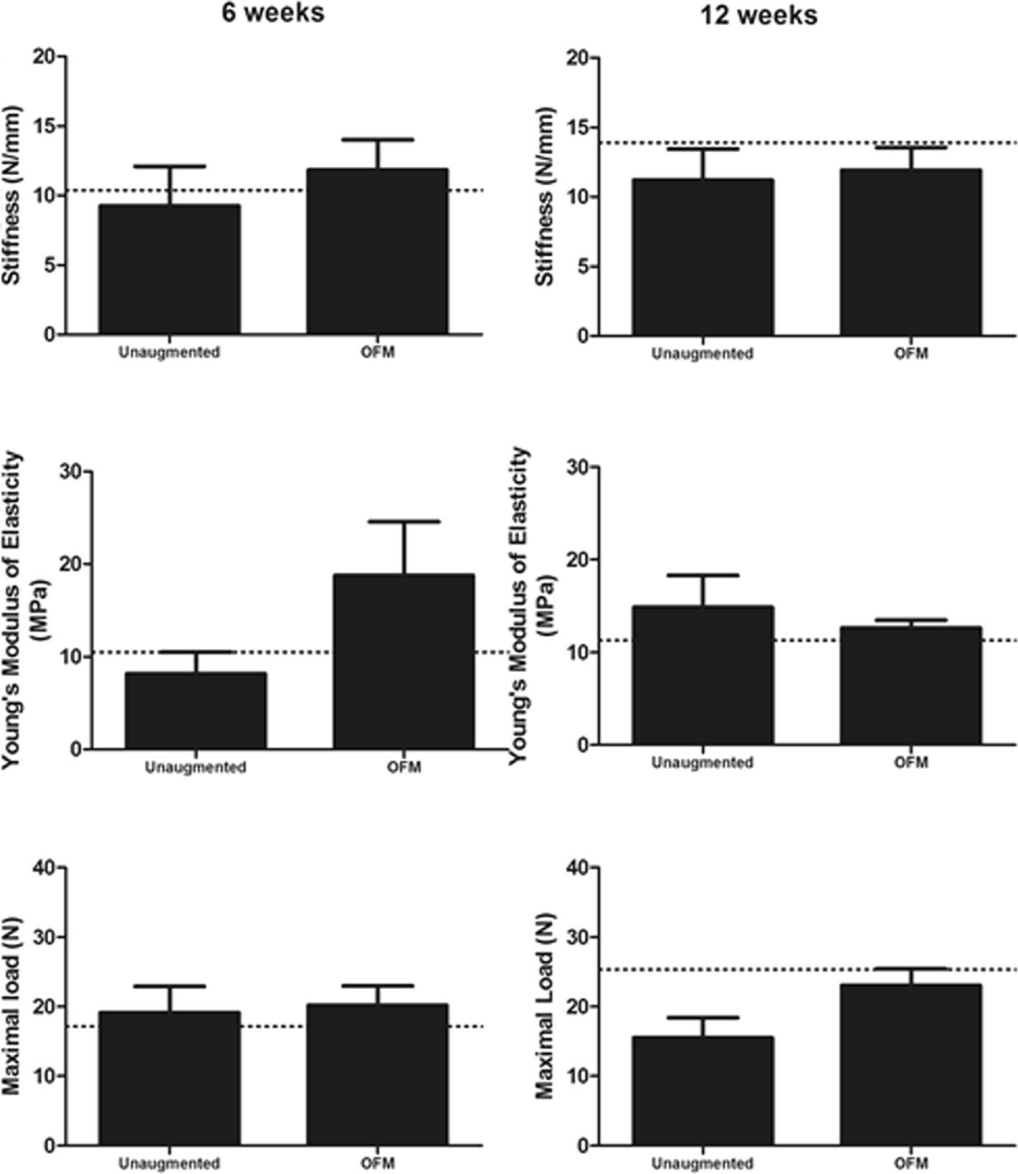
The biomechanical profiles of repaired rotator cuffs do not differ post-operatively. Mean stiffness, elasticity and maximal load to failure of unaugmented and OFM augmented rat shoulders at 6 and 12 weeks post-surgery. *Dotted line* indicates the mean measurements of sham-operated shoulders as a reference. *N* = 4

At 6 weeks post-repair, the stiffness and elasticity of the augmented rotator cuffs were higher than the unaugmented controls, although not significant (Fig. 4). Stiffness was measured at 11.8+/−2.2 N/mm in the augmented group, compared to 9.2+/−2.9 N/mm in the unaugmented group, and Young’s modulus of elasticity measured at 18.8+/−5.8 MPa for the augmented group and 8.2+/−2.3 MPa for the unaugmented group. Similarly, there was no significant difference between the unaugmented group and the sham group, which had a mean stiffness of 10.4+/−4.8 N/mm and a mean elasticity of 10.5+/−2.9 MPa.

At 12 weeks, there were similarly no differences in the stiffness and elasticity of all groups. Stiffness was measured at 11.9+/−1.6 N/mm for the augmented group, 9.1+/−2.2 N/mm for the unaugmented group and 13.9 N/mm for the sham-operated group. The Young’s modulus of elasticity measured was measured at 12.7+/−0.82 MPa for the augmented group, 14.9+/−3.4 MPa for the unaugmented group and 11.3 MPa for the sham-operated group.

There were no statistically significant differences in the load to failure of the repaired rotator cuffs. The mean load to failure was 20+/−2.8 N in the augmented group and 19+/−3.8 N in the unaugmented group at 6 weeks post-repair, and 23+/−2.4 N and 15+/−2.9 N at 12 weeks post-repair, respectively. The load to failure of the sham group was 17+/−3.8 N at 6 weeks and 25+/−6.6 N at 12 weeks.

### Histological analysis

At 6 weeks post-op, histological scoring of the repaired rotator cuffs showed no difference between the unaugmented group and the OFM augmented group, with both scoring 5.25 out of 15 (Table 2). The sham-operated group scored significantly higher at 11 out of 15 (Table 2).

**Table 2:** Histological grading of healed rotator cuffs, as scored by a specialist musculoskeletal pathologist and an orthopaedic registrar based on the criteria set out in Table 1

Group	Collagen fibre density	Collagen fibre orientation	Bone-tendon interface	Vascularity	Inflammation	Total
6 weeks						
Sham	2.5 (±0.29)	2.5 (±0.29)	2.5 (±0.29)	2.5 (±0.29)	2.5 (±0.29)	11 (±0.91)
Unaugmented	1.5 (±0.5)	0.75* (±0.48)	1 (±0.71)	1* (±0.41)	1* (±0)	5.25* (±1.60)
OFM augmentation	1.25 (±0.25)	1.25 (±0.25)	1 (±0.65)	0.25* (±0.25)	1* (±0)	5.25* (±0.85)
12 weeks						
Sham	2.75 (±0.25)	2.75 (±0.25)	2 (±0.71)	1.25 (±0.48)	2 (±0)	10.75 (±1.44)
Unaugmented	1.5 (±0.5)	0.5* (±0.29)	0.5 (±0.5)	0.5 (±0.29)	1.25 (±0.25)	4.25* (±1.60)
OFM augmentation	2 (±0)	1.25* (±0.25)	2 (±0.41)	0.25 (±0.25)	1.5 (±0.29)	7 (±1)

Data presented are the mean of both scorers ±SEM. One-way ANOVA with post hoc Tukey’s test. *N* = 2

*p < 0.05 compared to sham

**Fig. 5:**
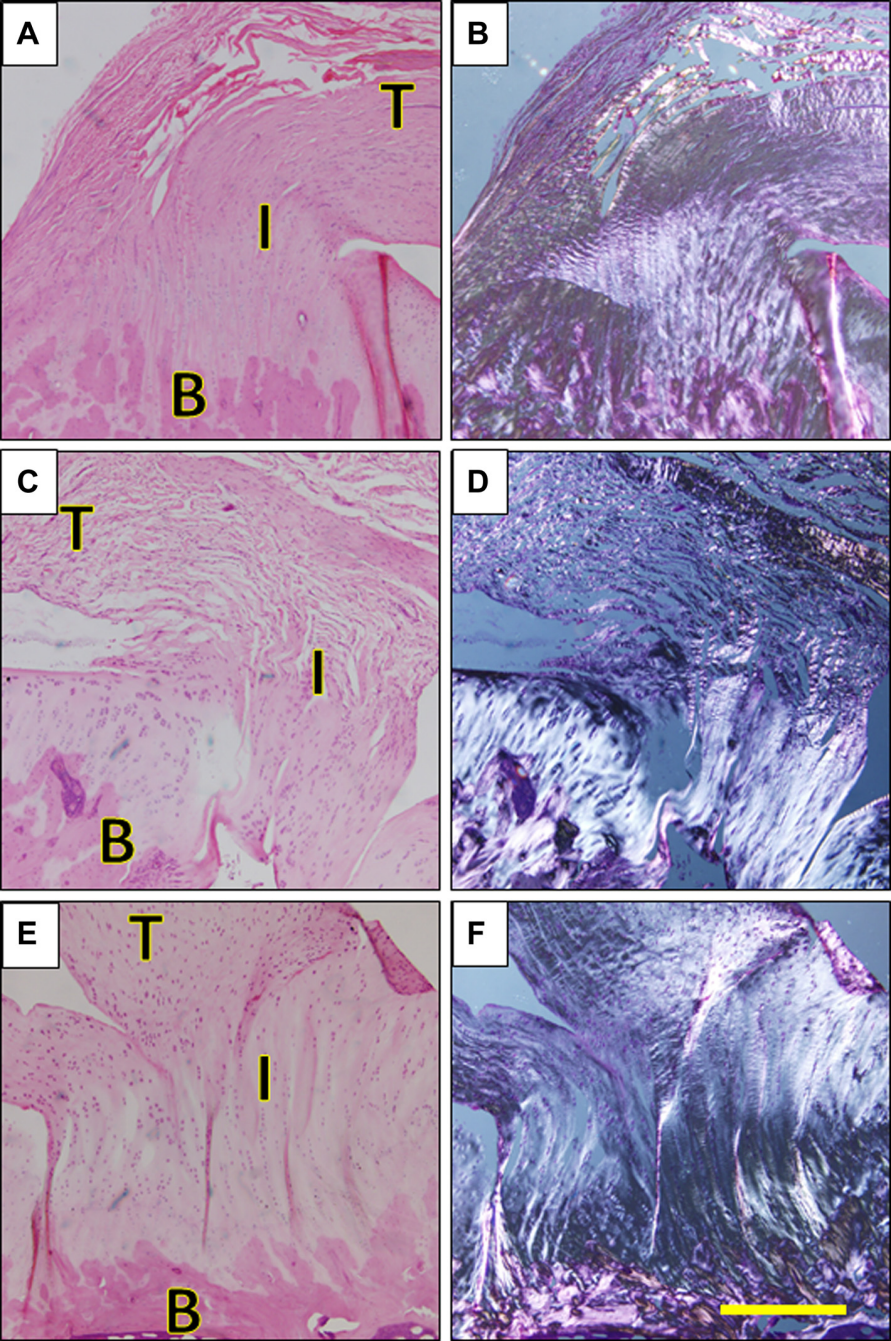
OFM improves healing of the tendon-bone interface. Representative micrographs of healing at the tendon-bone interface at 12 weeks post-operatively stained with haematoxylin and eosin and viewed under transmitted light (**a**, **c**, **e**) (*left*) and polarised light (**b**, **d**, **f**) (*right*). **a**, **b** Sham-operated shoulders. **c**, **d** Unaugmented control shoulders. **e**, **f** OFM augmented shoulders. Bone (*B*), tendon (*T*), tendon-bone interface (*I*). Scale bar represents 400 mm. *N* = 2

Histological analysis at 12 weeks post-op showed the sham-operated group to have a well-defined tendon-bone interface, with the collagen fibres of the tendon appearing dense and well organised (Fig. 5a, b); this was represented by a histological score of 10.75 (out of 15).

In the unaugmented group, while there was evidence of the initial stages of an intact tendon-bone interface, the tendon fibres appeared frayed and disorganised in nature (Fig. 5c, d), and they therefore scored significantly lower than the sham group in the histological scoring (4.25 out of 15).

The rotator cuffs that had been augmented with OFM had a well-formed tendon-bone interface and a dense collagen fibre network at the tendon; however, the fibres were largely disorganised and the tenocyte cells present had a rounded, non-tenocyte-like morphology (Fig. 5e, f). There was no significant difference in the histological score of the OFM group (7 out of 15) compared to either the sham group or the unaugmented group.

## Discussion

Here, we have shown that the novel ovine forestomach ECM scaffold material is cytocompatible to tenocyte cell growth, non-immunogenic and improves the histological appearance of the rotator cuff when used as an overlay augment in a pre-clinical model of rotator cuff repair.

Tears of the rotator cuff are a common, often painful and a debilitating problem [1]. Current rotator cuff repairs aim to re-attach the tendon to the humeral head and debride a partial tear or suture a full thickness tear using open or arthroscopic surgeries. While these techniques can be successful, disorganised or incomplete healing is common [7–9].

Previously, autografts from the biceps tendon or fascia lata have been trialled as interpositional grafts for improving rotator cuff repair surgical outcomes [25, 26] as have allograft rotator cuffs [27]. However, these methods have had varying results and have not been adopted as common practice. Biomaterial scaffold augmentation has therefore been suggested as a means to enhance the healing outcomes for small to large rotator cuff tears (grade 3 or 4) or when repairing a degenerative tendon [10].

According to a recent study, there were 13 FDA-approved biomaterial scaffolds available for use in augmenting rotator cuff repair. Of these, nine were ECM-based materials, three were synthetic and one was an ECM-woven polymer hybrid [10]. There are limited clinical studies on these materials, some suggesting that these scaffolds improve clinical outcomes; however, the results are generally inconclusive due to a lack of appropriate controls [28–30]. This leads to the consensus that currently available scaffolds, while promising, fail to meet clinical needs [10].

ECM-based scaffolds have been produced from many sources including dermis, small intestinal submucosa, pericardium, Achilles tendon and fascia lata of human, bovine, porcine or equine origin [11, 31]. OFM is an ECM scaffold produced from ovine forestomach. Cellular debris is removed using a novel decellularisation technique that maintains more of the natural structure of the ECM and thus improves the hosts healing response [17]. Previous studies have shown that OFM is non-toxic, supports mammalian cell growth and differentiation in vitro and enhances angiogenesis ex vivo and in vivo [17, 32]. Here, we have similarly shown that OFM supports the adherence and growth of primary tenocyte cells in vitro, although there did appear to be some variance between repeat experiments.

OFM has previously been used successfully in clinical and in vivo studies of wound healing [18–20, 22]; however, given the history of ECM-based materials in producing hypersensitivity reactions in patients undergoing rotator cuff repair [14–16], we feel that prior to pre-clinical animal models, it is important to assess the immunogenicity of novel biomaterials. Previous studies have demonstrated that maturation of monocyte-derived dendritic cells in response to biomaterials is indicative of in vivo inflammatory responses [33–35]. In this study, we demonstrated that OFM does not enhance the maturation of primary human dendritic cells in vitro and is therefore unlikely to illicit an adverse response when implanted in vivo.

In order to provide translational evidence that a novel material is a suitable scaffold, capable of improving the surgical outcomes of rotator cuff repair, pre-clinical assessment is required in a well-defined animal model of rotator cuff repair. Many animal models have been used to investigate rotator cuff pathology and repair [36]. Unlike the rabbit or sheep, the rat has a supraspinatus tendon enclosed under a bony arch, with a wide range of motion similar to that of humans, making the rat the recommended model for such procedures [36]. Both extrinsic and intrinsic insults to the supraspinatus tendon cause histological and biomechanical deterioration [4, 36], allowing the model to be used to mimic a wide range of rotator cuff pathologies and to investigate novel treatments for these pathologies. Here, we found the rat model to be relatively simple and reproducible, as well as practically and financially viable.

Augmentation with a biomaterial scaffold aims to both provide mechanical support to the repair and improve the quality of tendon healing [11]. In this study, there were no differences in the biomechanical properties (stiffness, elasticity and load to failure) between the unaugmented or OFM augmented rotator cuffs. The single-ply lyophilized OFM, used in this study, has relatively low mechanical strength compared to the native tendon (load to failure of 15.07 N and suture pull-out of 5.91 N). However, OFM biomaterial has been fabricated into multi-ply presentations, with load to failures of over 65 N [37], allowing scope for a more mechanically robust augment through tailoring of the fabrication procedure. However, these were not practical to use in this study due to the relative size of the rat supraspinatus.

Using overlay augmentation with the OFM scaffold, we showed improved histological outcomes compared to animals treated with standard surgical repairs and no significant difference in histological scoring between the OFM augmented tendons and the sham controls. Augmented tendons had improved collagen fibre density and orientation, and improved quality of healing at the tendon-bone interface. Importantly, at neither time points was there evidence of a hyper-inflammatory response to OFM, as shown by low vascularity and a low presence of inflammatory cells.

Previous studies have demonstrated that ECM-based materials induce healing responses by directing macrophage response down an M2, pro-remodelling phenotype, rather than the pro-inflammatory M1 phenotype [38]. Given the improvements in collagen fibre organisation and the lack of inflammatory responses seen in the histological sections, it would be of interest to determine what effect OFM has on macrophage response in tendon repair, although this was out of the scope of the present study. Similarly, determining how human tenocytes respond to OFM would be an important step in future translation from bench to bedside. Here, rat tenocytes were used as these are directly relevant to the pre-clinical model being employed.

## Conclusion

In conclusion, there is a clear clinical need for improving surgical outcomes of rotator cuff repair; however, the ideal biomaterial augment has not been identified or clinically implemented as yet. Here, we have shown that OFM as an overlay augment improves the histological appearance of the rotator cuff post-surgical healing but does not provide any mechanical benefit in its current iteration.

Overall, ECM scaffolds offer a promising solution to a difficult clinical problem. Identifying materials that offset the poorer mechanical properties of the rotator cuff post-injury/repair and enhance organised tendon healing still remain paramount to incorporating augmentation into current rotator cuff surgical procedures.
